# Anti-allodynic effect induced by curcumin in neuropathic rat is mediated through the NO-cyclic-GMP-ATP sensitive K^+^ channels pathway

**DOI:** 10.1186/s12906-020-2867-z

**Published:** 2020-03-14

**Authors:** Tracy Pastrana-Quintos, Giovanna Salgado-Moreno, Julia Pérez-Ramos, Arrigo Coen, Beatriz Godínez-Chaparro

**Affiliations:** 1 Departamento de Sistemas Biológicos, División de Ciencias Biológicas y de la Salud, Universidad Autónoma Metropolitana-Xochimilco, Calzada del Hueso 1100, Colonia Villa Quietud, 04960 Mexico, D.F., Mexico; 2 Departamento de Matemáticas, Facultad de Ciencias, Universidad Nacional Autónoma de México, CDMX, Apartado Postal 20-726, 01000 México, Mexico

**Keywords:** Curcumin, Neuropathic pain, Nitric oxide, SNL, Intrathecal administration

## Abstract

**Background:**

Recent studies pointed up that curcumin produces an anti-nociceptive effect in inflammatory and neuropathic pain. However, the possible mechanisms of action that underline the anti-allodynic effect induced by curcumin are not yet established. The purpose of this study was to determine the possible anti-allodynic effect of curcumin in rats with L5-L6 spinal nerve ligation (SNL). Furthermore, we study the possible participation of the NO-cyclic GMP-ATP-sensitive K^+^ channels pathway in the anti-allodynic effect induced by curcumin.

**Methods:**

Tactile allodynia was measured using von Frey filaments by the up-down method in female Wistar rats subjected to SNL model of neuropathic pain.

**Results:**

Intrathecal and oral administration of curcumin prevented, in a dose-dependent fashion, SNL-induced tactile allodynia. The anti-allodynic effect induced by curcumin was prevented by the intrathecal administration of L-NAME (100 μg/rat, a non-selective nitric oxide synthase inhibitor), ODQ (10 μg/rat, an inhibitor of guanylate-cyclase), and glibenclamide (50 μg/rat, channel blocker of ATP-sensitive K^+^ channels).

**Conclusions:**

These data suggest that the anti-allodynic effect induced by curcumin is mediated, at least in part, by the NO-cyclic GMP-ATP-sensitive K^+^ channels pathway in the SNL model of neuropathic pain in rats.

## Background

Neuropathic pain is defined as the pain arising as a direct consequence of a lesion or disease affecting the somatosensory system [1]. The prevalence of neuropathic pain in the general population is estimated at 3–17% [2]. Neuropathic pain could result from different etiologically disorders affecting the peripheral or the central neurvous system such as metabolic disorders (diabetes), viral infections (post-herpetic neuralgia, HIV, leprosy), neurodegenerative (Parkinson), autoimmune diseases (multiple sclerosis and Guillain-Barre syndrome), a tomor, trauma, esposure to toxinsor hereditary disease [3]. Treatment of neuropathic pain is based on tricyclic antidepressant (amitriptyline), gabapentinoids (gabapentin and pregabalin) and selective serotonin-norepinephrine reuptake inhibitors (duloxetine and venlafaxine) as the first-line treatment. Lidocaine, capsaicin, and tramadol have been proposed as the second-line treatment, while morphine, oxycodone and botulinum Toxin-A were included as third-line treatments for neuropathic pain [1]. Unfortunately, the treatment for neuropathic pain is inadequate due to poor drug efficacy and tolerability. Therefore, it is necessary to study more alternative therapies to mitigate neuropathic pain.

Curcumin [1,7-bis (4-hydroxy-3-methoxyphenyl)hepta-1,6-diene-3,5-dione] is an organic compound from the rhizome of the Indian spice turmeric (*Curcuma longa*), which is one of the principal ingredients in curry powder [4]. Its wide spectrum of biological activities including antiviral [5], antioxidant [6], neuroprotective [7], antidepressant [8], and anti-inflammatory effects [9]. In addition, curcumin has shown an anti-nociceptive effect in different types of pain, such as inflammatory pain [4, 7, 10], visceral pain [11], musculoskeletal pain [12], burning pain [13], and neuropathic pain [14–17]. Curcumin may alleviate neuropathic pain through inhibiting the expression of CX3CR1 by the activation of NF-κβ p65 in the dorsal horn of the spinal cord and dorsal root ganglion (DRG) [16]. Moreover, curcumin reversed the development of mechanical allodynia suppressing the activation of ERK and JNK in the spinal DRG [15]. Also, curcumin has an anti-allodynic effect through the noradrenergic and serotonergic systems by activation of the β_2_-adrenoceptor and 5-HT_1A_ receptor, respectively [17]. Curcumin decreased calcium ion accumulation in the sciatic nerve, decreased nitric oxide (NO) and lipid peroxidation (LPO), and increased endogenous antioxidant enzymes in vincristine-induced neuropathic pain [18]. Several lines of research indicated that NO induces analgesia and also that it mediates the peripheral and central anti-nociceptive effect of analgesic compounds, such as opioids, non-steroidal anti-inflammatory drugs, and natural products [19]. Other studies have reported that drugs which activate the NO-cGMP pathway seem to modulate the opening of the K^+^ channels in order to produce nociception [20]. Previous studies have indicated that natural products produce anti-nociceptive and anti-allodynic effects through the NO-cGMP-ATP sensitive channels K^+^ pathway [20–26]. There is evidence that suggests that curcumin exhibits its anti-nociceptive effect by directly stimulating K^+^ ATP channels in an inflammatory pain model [27]. Therefore, this work was undertaken to determine the possible anti-allodynic effect of curcumin in rats with spinal nerve ligation (SNL) model of neuropathy. Moreover, we investigated whether, at the central level, the NO-cGMP-ATP sensitive channels K^+^ pathway participates in the anti-allodynic effect induced by curcumin.

## Methods

### Animals

All experiments were performed on female Wistar rats weighing 140–160 g (*n* = 162). Previous studies have demonstrated no difference in tactile allodynia between female and male rats in the SNL model [23, 28, 29]. For this reason, we decided to use female rat in this study. The animals were provided by our bioterium and kept in isolated cages; rats were maintained with food (Lab Diet 5001) and water ad libitum. They were housed in groups at 22 ± 2 °C under 12:12 light-dark cycles. All experimental protocols were approved by the Research Bioethics Committee of the UAM-X. Animals were cared for the according to the current procedure for the Care and Use of Laboratory Animals (NOM-062-ZOO-1999, Mexico), and by the Guidelines on Ethical Standards for Investigation of Experimental Pain in Animals [30]. The rats were acclimatised to laboratory condition for 1 week prior to experiments, and the experiments were conclucted at 9:00 to 14:00. At the end of the experiments, rats were euthanised in a CO_2_ chamber.

### L5-L6 spinal nerve ligation model

To induce neuropathic pain, left L5 and L6 spinal nerve were performed as describe Kim and Chung (1992). Rats were anesthetized with a mixture of Ketamine (45 mg/kg, i.p) + Xylaxine (12 mg/kg, i.p.), a longitudinal skin incision was made on the left side of the spinal L4 to L6 level. Afterward, L5 and L6 spinal nerve were isolated and tightly ligated with 4–0 silk suture distal to the dorsal root ganglion. In sham surgery was concluded exposing L5 and L6 nerves but not ligated. Incisions were closed, and rats were allowed to recover for 14 days [31].

### Measurement of anti-allodynic activity

Tactile allodynia was evaluated by measuring paw withdrawal threshold. The von Frey filaments were applicated vertically for 10 s to the plantar surface on the right hind paw. The tactile allodynia was determinated by a positive response such as an abrupt widrawal hind paw. The cut-off value was a negative response to 15 g. the paw withdrawal threshold was determinated using the up-down method with an application of a series of consefutive von Frey filaments (0.4, 0.7, 1.2, 2.0, 3.6, 5.5, 8.5, and 15 g). The resulting scores were used to calculate the 50% response threshold using the formula:

$$ 50\%\kern0.35em g\kern0.35em threshold={10}^{\left({X}_f+\upkappa \updelta \right)}/10,000. $$

where X_f_ = the value (in log units) of the final von Frey filament used, κ = the value from the table for the pattern of positive and negative responses published previously by Chaplan and co-workers (1994), and δ = the mean difference (in log units) between stimuli [32]. The 50% threshold withdrawal was assessed before and at 0, 30, 60, 120 and 240 min after drug administration. Allodynia was considered present when the paw withdrawal threshold was < 4 g [32, 33]. Animals with a basal withdrawal threshold above 4 g or that showing motor deficiency were not included in the experiments.

### Drugs

Curcumin (C1386), glibenclamide (G0639), N-nitro-L-arginine methyl ester (L-NAME, N5751), 1H -[1, 2, 4] oxadiazolo [4,3-a]quinoxaline-1-one (ODQ, O3636), NaOH (S5881), and Dimethyl sulfoxide (DMSO, 67–68-5) were purchased from Sigma Aldrich (St. Louis, MO, USA). Curcumin used for per oral (p.o.) administration was dissolved in NaOH to 0.5 M into a volume of 10 ml/kg. Moreover, curcumin and glibenclamide were dissolved in 10% DMSO, and ODQ and L-NAME were dissolved in isotonic saline.

### Lumbar puncture

The rats were anaesthetized with 2% isoflurane and received an intrathecal injection by lumbar puncture 14 days after spinal nerve ligation of L-5/L6, as previously reported by Mestre and co-workers (1994). The animals were holding with one hand at the pelvic girdle level and the drug was injected intrathecally with a 30-G needle connected to a 25-μl Hamilton syringe in the intrathecal space between the L5 and L6 vertebrae on the dorsal side and perpendicular to the vertebral column [34].

### Study design

In order to determine the possible anti-allodynic effects of curcumin on neuropathic pain, we used 162 neuropathic rats that were randomly divided into the following groups: SNL (*n* = 6) and sham (*n* = 6) animals received 20 μl of a vehicle (saline at 0.9%) via intrathecal administration (i.t.) or increasing doses of curcumin via i.t. (30, 100, 200, and 300 μg/rat; *n* = 6 per group). On the other hand, SNL (*n* = 6) and sham (*n* = 6) animals received an oral (2 ml) administration of vehicle (carboxymethylcellulose) or increasing doses of curcumin (10, 100, 310 mg/kg, p.o.); *n* = 6 per group). In both cases, the effect of curcumin on the paw withdrawal threshold was evaluated by the up-down method at 0, 30, 60, 120, and 240 min after intrathecal or oral administration. The doses and drug administration schedules for curcumin were selected based on a pilot experiment in our laboratory. Intrathecal (100 μg/rat, *n* = 6, [35]) or oral (100 mg/kg, *n* = 6, [36]) gabapentin was used as a positive control.

In order to determine the possible participation of the NO-cGMP-ATP-sensitive K^+^ channel pathway in the curcumin-induced anti-allodynic effect, we administrated the nonselective NO synthase inhibitor L-NAME in doses of (10 and 100 μg/rat; *n* = 6, [37]), the guanylyl cyclase inhibitor ODQ in doses of (1 and 10 μg/rat; *n* = 6, [37]), and an ATP-sensitive K^+^ channel blocker glibenclamide in doses of (5 and 50 μg/rat; *n* = 6, [37]).The anti-allodynic effect of the co-administration of antagonists and curcumin was evaluated at 0, 30, 60, 120, and 240 min after administration.

### Data statistical analysis

In the experiments using the von Frey withdrawal threshold, curves were constructed as the mean ± S.E.M plotting the 50% withdrawal threshold as a function of time. Moreover, to analyse the effect of the different treatment, the data were normalized by calculating the area under the 50% withdrawal threshold against time curves (AUC). The area under the 50% withdrawal threshold against the time curve (AUC) was calculated by the trapezoidal rule. The percentage of maximum possible effect (%MPE) was calculated with the following formula:

$$ \%\mathrm{MPE}=\frac{\ \mathrm{AUC}\ \mathrm{Drug}-\mathrm{AUC}\ \mathrm{Vehicle}}{\mathrm{AUC}\ \mathrm{Sham}-\mathrm{AUC}\ \mathrm{Vehicle}}\times 100. $$

Statistical differences between groups were determined by one-way analysis or two-way repeated measures analysis of variance followed by a post-hoc test; we applied Tukey’s post-hoc test for one-way analysis experiments and Bonferroni post-hoc test for two-way analysis experiments. Differences were considered as statistically significant when *P* ≤ 0.05.

## Results

**Fig. 1:**
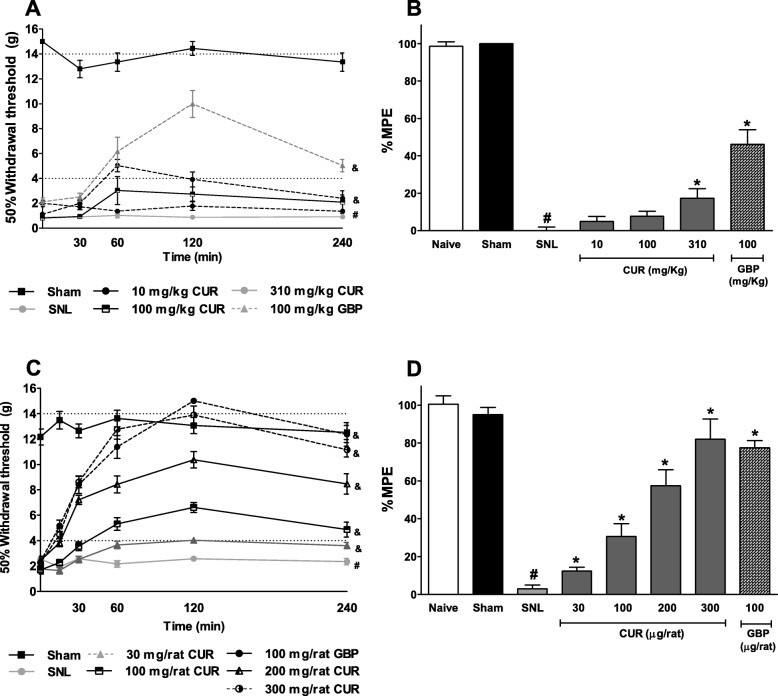
The time course of the anti-allodynic effect following oral (**a**) and intrathecal (**c**) administration of curcumin (CUR) in rats who underwent to L5-L6 SNL. The withdrawal threshold was determined 14 days after surgery. The bars show the maximum possible effect (%MPE) (**b** and **d**). Data are expressed as mean ± S.E.M. of six animals. *P ≤ 0.05 versus SNL group and ^#^P ≤ 0.05 versus sham group was determined by one-way ANOVA followed by Tukey test. ^&^P ≤ 0.05 versus SNL group, determined by two-way repeated measures analysis of variance followed by the Bonferroni test. Abbreviations: GBP: Gabapentin, Veh: vehicle; SNL: spinal nerve ligation

14 days after the ligation of the L5-L6 spinal nerves (SNL), the basal values of the 50% paw withdrawal threshold was disminished (≤ 4 g) in the ipsilateral paw as compared to sham group (15 g), indicating that the ligation of the L5-L6 spinal nerves induced tactile allodynia. It was observed from days 1 to 14 (Fig. 1a). Fourteen days after the SNL surgery, oral administration with curcumin 310 mg/kg, but not the lower doses (10 and 100 mg/kg) or vehicle, significantly reversed the tactile allodynia in neuropathic rats (Fig. 1a, F_12,80_ = 12.9, *P* < 0.0001). In addition, intrathecal administration with curcumin, but not the vehicle, significantly reversed the allodynia in SNL rats in a dose-dependent fashion (Fig. 1c, F_15,100_= 25.74, *P* < 0.0001). Oral (100 mg/kg) and intrathecal (100 μg/rat) administration with an effective gabapentin dose, used as a positive control, significantly reversed tactile allodynia in 46.05 ± 7.9% and 77.5 ± 3.7%, respectively (Fig. 1). The dose of 310 mg/kg curcumin (p.o.) had 17.2 ± 2.2% of the maximum possible anti-allodynic effect (%MPE) compared with 46.05 ± 7.9% of the %MPE induced by gabapentin (Fig. 1b, F_6, 30_ = 167.0, *P* < 0.0001). Moreover, intrathecal administration with curcumin (300 μg/rat, i.t.) produced a maximal effect of about 82.0 ± 4.3%, whereas gabapentin produced an efficacy of 77.5 ± 3.7% (Fig. 1d, F_7,38_ = 244.6, *P* < 0.0001). In both cases, the maximal anti-allodynic effect occurred 120 min after the oral (Fig. 1a) or intrathecal (Fig. 1c) administration of curcumin and gradually declined in about 4 h.

### Effect of L-NAME, ODQ, and glibenclamide on curcumin-induced anti-allodynic effect

**Fig. 2:**
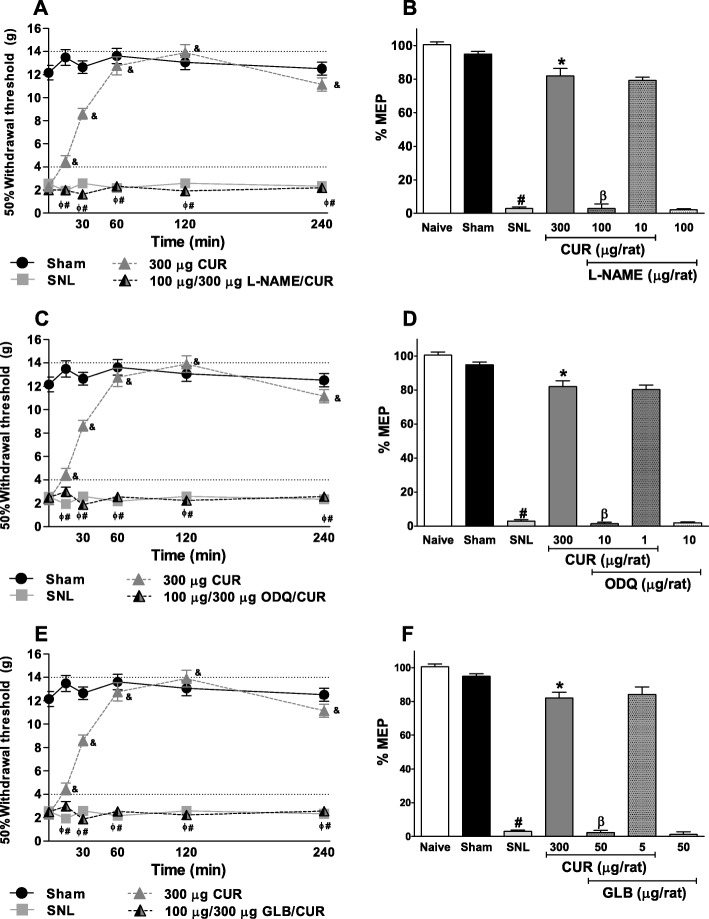
Time course of the effect of intrathecal pretreatment with the inhibitor of oxide nitric synthase, L-NAME (**a**); the selective inhibitor of guanylate cyclase soluble, ODQ (**c**); and the blocker of ATP sensitive K^+^ channels, glibenclamide (GLB) (**e**); on the anti-allodynic effect induced by curcumin (CUR) in rats who underwent to L5-L6 spinal nerve ligation (SNL). Data are expressed as mean ± S.E.M. of six animals. The withdrawal threshold was determined 14 days after surgery. The bars show the maximum possible effect (%MPE). *P ≤ 0.05 versus SNL group and ^#^P ≤ 0.05 versus Sham group and ^β^P ≤ 0.05 versus CUR, determined by one-way ANOVA followed by Tukey test. ^&^P ≤ 0.05 versus SNL group and ^Ф^P ≤ 0.05 versus CUR group was determined by two-way repeated measures analysis of variance followed by the Bonferroni test. Abbreviations: L-NAME: Nω-nitro-L-arginine methyl ester; ODQ: 1H -[1, 2, 4] oxadiazolo [4,3-a]quinoxalin-1-one; SNL: spinal nerve ligation

Intrathecal pre-treatment with L-NAME (100 μg/rat, Fig. 2a, F_15,100_ = 26.36, *P* < 0.0001 and 2b, F_6,34_ = 422.8, *P* < 0.0001), ODQ (10 μg/rat, Fig. 2c, F_15,100_ = 27.84, *P* < 0.0001 and 2d, F_6,35_ = 583.2, *P* < 0.0001) or glibenclamide (50 μg/rat, Fig. 2e F_15,100_ = 27.06, *P* < 0.0001 and 2f, F_6,34_ = 421.9, *P* < 0.0001), significantly prevented the anti-allodynic effect of curcumin (300 μg/rat) in neuropathic rats. Tukey’s test also confirm these differences between treatment groups (Fig. 2a, c, and e). The administration of L-NAME, ODQ, and glibenclamide per se did not affect the allodynia induced by L5-L6 SNL (Fig. 2).

## Discussion

Acute intrathecal administration of curcumin, but not oral administration, reversed, in a dose-dependent fashion, established tactile allodynia in SNL rats. These results were consistent with previous studies which demonstrated that acute or chronic intrathecal administration of curcumin reversed allodynia and hyperalgesia induced by L5-L6 spinal nerve ligation, chronic constriction injury, or in complete Freund’s adjuvant models [4, 38]. Additionally, chronic, but not acute, oral curcumin treatment alleviated mechanical allodynia and thermal hyperalgesia in the neuropathic pain model [17]. Moreover, curcumin attenuated the CFA-induced mechanical allodynia and heat hyperalgesia [4]. Taken together, these data suggest that curcumin could be useful to relieve neuropathic pain in rats.

### Curcumin-induced anti-allodynic effect following administration of L-NAME, ODQ, and glibenclamide

Previous studies have suggested the possible role of the NO-cGMP pathway in activating targets such as potassium channels [19]. The opening of K^+^ channels due to the L-arginine-NO-cGMP pathway allows regulation of neuronal excitability through K^+^ ions permeating the membrane [39]. Furthermore, several studies have shown that the opening of K^+^ channels induces anti-nociception [36, 40]. In order to study whether curcumin leads to an increase of NO synthesis, L-NAME, a non-selective NOS inhibitor was utilised, blocking the synthesis of NO [36]. It was observed that L-NAME prevented the spinal anti-allodynic effect induced by curcumin in a dose-dependent fashion, suggesting that NO is an important mediator. In addition, the administration of ODQ, an inhibitor of the soluble guanylyl cyclase enzyme, prevented, in a dose-dependent fashion, the spinal anti-allodynic effect of curcumin. Taken together, our data suggest that curcumin is able to induce the anti-allodynic effect by increasing NO and cGMP production. Nevertheless, some reports indicate that curcumin inhibits NO production with concomitant down-regulation of iNOS mRNA in LPS-activated RAW 264.7 macrophages and LPS-stimulated microglia cell [41, 42]. However, several studies indicate that the NO/cGMP signalling cascade has either anti - or pro-allodynic effects in neuropathic pain models, as well as opposing effects in peripheral inflammatory models [19, 41, 43]. The different effects of the NO-cGMP signal cascade concerning noxious transduction may be due, at least in part, to differences in experimental conditions. In addition, the intrathecal glibenclamide administration prevented the anti-allodynic effect of curcumin in the L5-L6 spinal nerve ligation model. These results suggest that curcumin produces an anti-allodynic effect by activating K^+^ ATP-sensitive channels. According to these findings, previous observations of De Paz-Campos and co-workers (2012) show that curcumin is able to produce anti-nociceptive effects through activation of K^+^ ATP sensitive channels in an inflammatory model of pain [27]. Taken together, our data suggest that curcumin produces an anti-allodynic effect by activation of NO-cGMP ATP- sensitive K^+^ channels pathways.

## Conclusion

Oral and spinal administration of curcumin reduced tactile allodynia in neuropathic rats. The anti-allodynic effect induced by curcumin was prevented by L-NAME, ODQ, and glibenclamide. These findings suggest that curcumin extenuates the allodynia through the involvement of NO-cGMP-ATP-sensitive K^+^ channel pathways, suggesting that curcumin could be useful for the treatment of neuropathic pain.

## Data Availability

The datasets analyzed during the current study are available from the corresponding author on reasonable request.

## Abbreviations

%MPE: Percentage of maximum possible effect
5-HT_1A_: Serotonin 1A receptor
ATP: Adenosine triphosphate
AUC: Area under curve
CFA: Complete freund’s adjuvant
cGMP: Cyclic guanosine monophosphate
CO_2_: Carbon dioxide
CX3CR1: CX3C chemokine receptor 1
DMSO: Dimethyl sulfoxide
DRG: Dorsal root ganglion
ERK: Extracellular-signal-regulated kinase
F: F-test
HIV: Human immunodeficiency virus
i.t.: intrathecal administration
iNOS: inducible nitric oxide synthase
JNK: c-Jun N-terminal kinase
K^+^: Potassium
Kg: Kilogram
L5-L6: Lumbar 5 – Lumbar 6
L-NAME: N-nitro-L-arginine methyl ester
LPO: Lipid peroxidation
LPS: Lipopolysaccharides
ml: milliliter
mRNA: messenger Ribonucleic acid
NaOH: Sodium hydroxide
NF-κβ: Nuclear factor kappa-light-chain-enhancer of activated B cells
NO: Nitric oxide
o.p.: oral administration
ODQ: 1H-[1, 2, 4] oxadiazolo [4,3-a]quinoxaline-1-one
p: *p*-value
S.M.E.: Standard error of the mean
SNL: Spinal Nerve Ligation
μl microliter;°C: Degree centigrade
